# La Belle Alliance

**Published:** 1905-04-08

**Authors:** 


### La Belle Alliance.

The proposed return visit of English physicians and surgeons to Paris has now taken a definite shape. An important committee of patronage has been formed in Paris with an executive committee which, with true French powers of organisation, has grouped itself into a series of sections representative of every branch of medicine and surgery. The date of the visit is fixed to begin upon Wednesday, May 10th, and on that evening the English guests will be received at the Sorbonne by the Rector of the University of Paris and by M. Casimir-Périer, who is president of the "Amis de l'Université." As was the case in London, every facility will be afforded for seeing the work of the medical institutions for which Paris is celebrated, and those who do not already know them will be able to imagine the advance which has taken place since Claude Bernard worked in a cellar at the Collège de France, when Wurtz had only a lumber-room in the attics of the Dupuytren Museum, and when J. B. Dumas, the chemist, had such unhealthy rooms allotted to him at the old Sorbonne that he has been obliged to maintain a laboratory at his own expense. Yet the work done in these holes was marvellous in character, and it is to be doubted whether, in the magnificent surroundings of the present day, men produce a tithe of what was then given to the world by the giants of the last generation. A banquet will conclude the visit on Saturday evening, and it is proposed that if sixty members give in their names before April 30th there will be an excursion to Vichy on Monday, May 15th, which will be so arranged that the whole expense will be borne by the promoters. It seems at the present time that about one hundred and seventy members of the medical profession will attend the meeting in Paris, and they will be accompanied by thirty ladies.

The connection between the French and British schools of medicine was much closer at the beginning of the last century than it is at present. Few of the better class of medical students were then content to settle in practice until they had spent some time in Paris, though the journey was long and costly. Thus, Sir Robert Christison, in 1820, left London for Brighton on the outside of a splendidly horsed four-in-hand stage coach. At Brighton, after much trickery and squabbling among the seafaring people on the beach, he was carried out to the Dieppe packet, only a quarter of a mile from the shore, in a leaky boat carrying twelve passengers, at the exorbitant charge of three shillings a head. He says: "Nothing could be worse than the treatment we met with on this passage. We paid the Custom-house porters one shilling for not examining our trunks, three shillings to the rascally shoremen for rowing us on board, two guineas for our passage of about one hundred miles across the Channel, and ten shillings for our dietary of milkless tea, bread and putrid butter during our forty hours' voyage. Next morning we left Dieppe for Rouen, 45 miles, by the Diligence Célérifère, which was warranted to travel seven miles an hour. We slept at Rouen, and next day completed the journey to Paris, a distance of 90 miles." Now one leaves Charing Cross after lunch, dines on the way, and is in Paris shortly after nine o'clock, at a cost for a return ticket which would hardly have defrayed the sea passage eighty years ago. Many go to Germany and to Vienna to study, but comparatively few now stay in Paris; yet we have much to learn from the French, and it is by means of the goodwill shown at such gatherings that we appreciate how easily we can amend our own shortcomings in exactly those points wherein the French schools excel us. The invaluable work of Pasteur was turned to practical advantage by Lister; and if Pasteur's frail body had succumbed to his indomitable spirit when he had his apoplectic attack in October 1868, the English school of surgery would certainly never have occupied its present proud position. How wonderfully, too, has the work of Laennec, of Trousseau, and of Charcot fructified in the minds of English-speaking physicians!

